# Lactate Dehydrogenase A Expression Is Necessary to Sustain Rapid Angiogenesis of Pulmonary Microvascular Endothelium

**DOI:** 10.1371/journal.pone.0075984

**Published:** 2013-09-26

**Authors:** Glenda Parra-Bonilla, Diego F. Alvarez, Mikhail Alexeyev, Audrey Vasauskas, Troy Stevens

**Affiliations:** 1 Department of Pharmacology, University of South Alabama, Mobile, Alabama, United States of America; 2 Department of Medicine, University of South Alabama, Mobile, Alabama, United States of America; 3 Department of Cell Biology and Neuroscience, University of South Alabama, Mobile, Alabama, United States of America; 4 Department of Biochemistry and Molecular Biology, University of South Alabama, Mobile, Alabama, United States of America; 5 Center for Lung Biology, University of South Alabama, Mobile, Alabama, United States of America; Indiana University, United States of America

## Abstract

Angiogenesis is a fundamental property of endothelium, yet not all endothelial cells display equivalent angiogenic responses; pulmonary microvascular endothelial cells undergo rapid angiogenesis when compared to endothelial cells isolated from conduit vessels. At present it is not clear how pulmonary microvascular endothelial cells fulfill the bioenergetic demands that are necessary to sustain such rapid blood vessel formation. We have previously established that pulmonary microvascular endothelial cells utilize aerobic glycolysis to generate ATP during growth, a process that requires the expression of lactate dehydrogenase A to convert pyruvate to lactate. Here, we test the hypothesis that lactate dehydrogenase A is required for pulmonary microvascular endothelial cells to sustain rapid angiogenesis. To test this hypothesis, Tet-On and Tet-Off conditional expression systems were developed in pulmonary microvascular endothelial cells, where doxycycline is utilized to induce lactate dehydrogenase A shRNA expression. Expression of LDH-A shRNA induced a time-dependent decrease in LDH-A protein, which corresponded with a decrease in glucose consumption from the media, lactate production and cell growth; re-expression of LDH-A rescued each of these parameters. LDH-A silencing greatly reduced network formation on Matrigel *in vitro*, and decreased blood vessel formation in Matrigel *in vivo*. These findings demonstrate that LDH-A is critically important for sustaining the rapid angiogenesis of pulmonary microvascular endothelial cells.

## Introduction

Lung endothelium is generally thought to be quiescent in the healthy postnatal vasculature [1]. This concept has been disputed, as some investigators believe that there is a program for lung tissue homeostasis, referred as the lung structure maintenance program, in which cells within the alveolus continuously undergo apoptosis and proliferation [2]. The principle of the lung structure maintenance program is that such turnover is necessary to replace injured or senescent cells [2]; disruption of this delicate balance results in vascular disease.

Pulmonary microvascular endothelial cells (PMVEC) form the vascular barrier of the alveolar-capillary membrane, and limit fluid filtration into the alveoli and interstitium, protecting the gas-exchange region [3,4,5]. Any disruption of the endothelium in this region must be promptly repaired, a process that may require rapid endothelial cell proliferation. In more severe cases, capillary repair involves an angiogenic response to replace lost vessels. Capillary endothelium undergoes robust angio-proliferative responses, both *in vivo* and *in vitro* [6,7,8,9]. PMVECs, for example, are rapidly angiogenic [10,11]. These cells proliferate more quickly than do pulmonary artery endothelial cells (PAECs), and they are enriched with a higher number of replication-competent cells. Some of the molecular components that contribute to the angio-proliferative phenotype of PMVECs have been elucidated, including nucleosome assembly protein-1 (NAP-1) [11], cyclin D1, cdk2 and cdk4 [12]. NAP-1 is an epigenetic factor implicated in transcriptional regulation, and cyclin D1 and cdk are involved in cell cycle control. Therefore, all of these proteins constitute key molecular fingerprints of PMVEC hyperproliferation.

We recently sought to determine how PMVECs sustain their bioenergetic needs during rapid proliferation, and observed that they utilize aerobic glycolysis to maintain ATP concentrations that are roughly 2-fold higher than in pulmonary artery endothelial cells [13]. Lactate dehydrogenase-A (LDH-A) expression was needed to consume glucose and generate lactic acidosis that paralleled rapid proliferation. It is not presently clear whether PMVECs similarly utilize aerobic glycolysis to support neo-angiogenesis. Our present studies therefore tested the hypothesis that LDH-A expression is necessary to sustain rapid neo-angiogenesis in PMVECs.

## Materials and Methods

### Ethics Statement

Animal studies were conducted according to guidelines, and following approval, of the University of South Alabama Animal Care and Use Committee.

### Isolation and culture of pulmonary endothelial cells

Cells used for this study were isolated and sub-cultured from male CD rats weighing 200-250 g using methods detailed previously [3,10,13].

### Generation of LDH-A knockdown using a Tet-Off system

**Figure 1:**
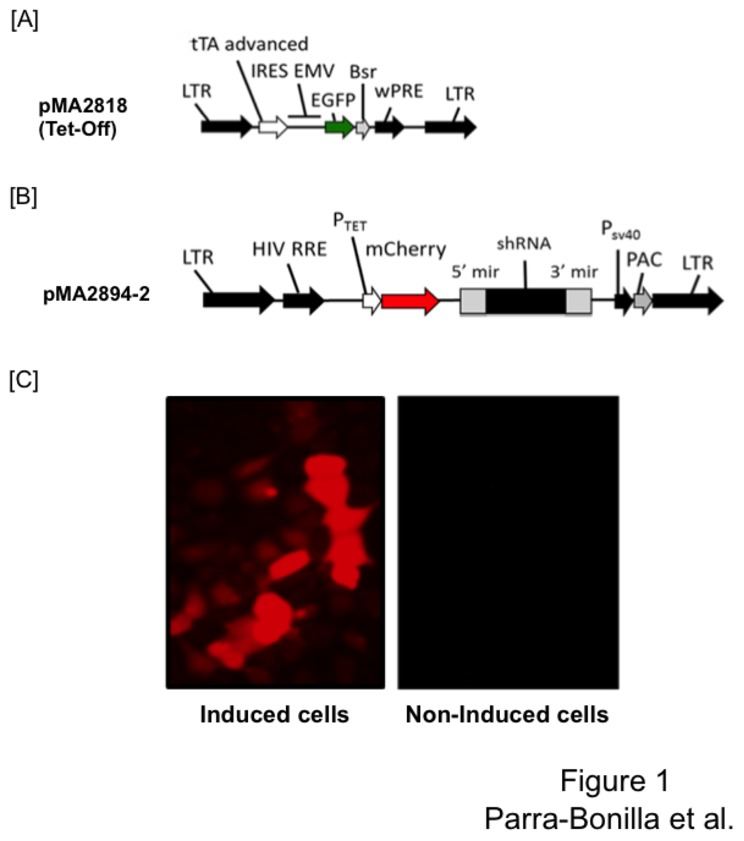
Map of the Tet-Off construct and induction of LDH-A shRNA. (A) PMVECs were infected with a retrovirus, enabling reverse tetracycline-controlled transactivator protein (tTA) expression. Cells were selected to homogeneity using blasticidin, reinfected with a lentivirus [shown in (B)], and selected to homogeneity using puromycin. The resulting double-transfection enabled the expression of a LDH-A short hairpin RNA (shRNA) in the absence of doxycycline. Bsr, blasticidin resistance gene; EGFP, enhanced green fluorescent protein; HIV RRE, human immunodeficiency virus Rev response element; IRES EMV, encephalomyocarditis virus internal ribosome entry site; LTR, retro/lentiviral long terminal repeat; PAC, puromycin resistance gene; PSV40, simian virus 40 promoter; PTet, doxycycline-regulated promoter; wPRE, woodchuck hepatitis virus posttranscriptional regulatory element; mir, 5’–3’ flanking sequence derived from the murine mir (micro-RNA gene)-15. (C) Doxycycline retrieval promoted expression of the red fluorescent protein mCherry in infected PMVECs.

Using a retro-lentiviral infection method [14], we generated a double transfected PMVEC line that expresses the tetracycline transactivator protein (tTA) and LDH-A shRNA (Figure **1A and 1B**). In the first step, cells were infected with a retrovirus encoding rTA and selected to homogeneity with blasticidin, and reinfected with lentivirus containing the shRNA. Cells were then selected with puromycin, and infected cells were grown and expanded in the presence of doxycycline (2 µg/ml). Cells were induced by doxycycline-withdrawal to allow the interaction of tTA with TRE, which promoted shRNA transcription. Transcription efficiency was assessed by the intensity of red fluorescent protein mCherry, which is transcribed along with LDH-A shRNA (Figure **1C**). The same shRNA sequence that we previously used to knockdown LDH-A in a Tet-On system was used to develop the Tet-Off system [13].

### Evaluation of the Tet-Off system to knockdown LDH-A

Growth curves with uninduced (with doxycycline) and induced (no doxycycline) cells containing the shRNA sequence that successfully inhibited LDH-A expression were performed [13]. Daily protein samples were collected for western blots as well as medium samples to measure glucose depletion from the media and lactate production. Rescue experiments were performed where cells were grown in the absence of doxycycline (induced cells) for 6 days, and then doxycycline was added to the media. During this experiment the same parameters were measured: cell number, protein levels, glucose depletion and lactate production.

### Western blots

Samples from the lentivirus-infected cells (PMVECs, Tet-Off cell line) were collected during log phase growth and used to extract total protein. Cells were lysed using a lysis buffer containing 1% SDS, 10% DMSO and 200 mM sodium acetate, plus a proteinase inhibitor cocktail. Protein concentration was quantified using a modified Lowry assay from Sigma and albumin was used to make a standard curve for the assay. Samples were boiled for 5 minutes, and equal amounts of total protein were loaded into precast SDS/PAGE 4-12% gradient gels from BioRad. The gels were run at 200 V for 1.5 hours. Proteins were transferred overnight at 4°C using nitrocellulose membranes from BioRad. Primary antibody from Santa Cruz Biotechnology was used for the immunoblotting of LDH-A (sc-27230) (1:200 dilution). After two hours of incubation with the primary antibody, the membrane was incubated with a secondary antibody conjugated with horseradish peroxidase (1:5000). Finally, a pico-western detection kit from Pierce (SuperSignal West Pico) was used to visualize the bands.

### Network formation on Matrigel *in vitro* using Tet-On and Tet-Off system

Tet-On (induced), Tet-Off (induced and non-induced) and control cells (wild type cells) were seeded on Matrigel (100 µl) in 48 well plates at a density of 3.5 x 10^4^ cells, and 200 µl of media was added [10]. Cells were incubated for 24 hours, and then observations made and pictures taken to report the presence or absence of networks [10].

### Angiogenesis assay *in vivo*: Matrigel plugs in rats

CD male rats (Charles River Laboratories) were anesthetized using isoflurane gas according to University of South Alabama Animal Care and Use Committee. Once an anesthetic plane was achieved, cells mixed with Matrigel were injected in the abdomen of the animals (approximately 5 minutes per rat) [10]. Cells engineered using Tet-On (non-induced cells) and Tet Off (induced cells) systems and control cell lines, including wild type PMVECs and cells engineered with Tet-On and Tet-Off constructs lacking LDH-A shRNA, were grown to confluence. Cells were trypsinized and counted. 3.75 x 10^5^ cells were suspended in 250 µl and mixed with 500 µl of un-polymerized Matrigel (BD laboratories) at the moment of the injection [10]. The mixture was injected subcutaneously into the left and right abdomen of the rats using a 23-gauge needle. Tet-On and Tet-Off cells were injected into the same rat to minimize variations between animals. Control cells and Matrigel alone without cells were randomly distributed in different animals. The Matrigel plugs were left in the animal for 10 days. Following the procedure rats were transferred back to their cages where they rapidly recovered.

### Removal of the Matrigel plugs

After 10 days, the animals were anesthetized using either ketamine 75 mg/kg of body weight or Nembutal (50 mg/kg of body weight) and the skin from the abdomen was incised to reveal the plugs [10]. Plugs were carefully removed and placed into plastic cassettes and immersion fixed in 10% buffered formalin. Pictures were taken from the un-processed plugs. Paraffin blocks were made from the fixed specimens and cut in 5 µm sections. These sections were used to make un-stained and hematoxylin and eosin (HE) stained slides. Anesthetized animals were euthanized by exsanguination.

### Analysis of slides

Hematoxylin and eosin stained slides were observed under the light microscope and pictures were taken. Sections were extensively examined to count the number of vessels, defined by lumen-containing red blood cells [10]. Surface area was calculated by taking pictures of the plugs at 2x magnification, in many cases more than two pictures were required to cover all of the surface area. The pictures were then used to draw the area of the plug to be analyzed with the imageJ software (NIH). Measured values were standardized to mm^2^ based on the value of a known area. The results were then reported as number of formed blood vessels per mm^2^.

### Immunohistochemistry

Unstained paraffin sections were heated to 60°C for 20 minutes and deparaffinized with three washes of xylene for 5 minutes each. Tissue was rehydrated using a gradual decrease in alcohol (100% and 95%) content for 5 minutes and two times each concentration. Slides were then immersed in distilled water for 5 minutes total. Antigen retrieval was performed by microwaving slides in pre-boiled 0.01 M sodium citrate buffer (pH 5.0) for 1 minute at low power followed by 2 minutes at room temperature. This process was repeated nine additional times. Slides were allowed to cool for 20 minutes at room temperature and washed once in distilled water for 5 minutes followed by three 5-minute PBST washes. Sections were incubated with 3% H_2_O_2_ for 10 minutes to suppress endogenous peroxidase activity followed by three 5-minute PBST washes. Slides were blocked for 1 hour in a humidified chamber at room temperature with blocking buffer: 5 mL PBST plus one drop (~50 µL) horse serum from VECTASTAIN. Then, a primary antibody for GFP (Ab290 from Abcam, used at 1:200) was diluted as noted in fresh blocking buffer and incubated overnight at 4°C in a humidified chamber. Slides were rinsed with PBST three times for 5 minutes each and then incubated with biotinylated horse anti-rabbit secondary antibody (VECTOR) for 45 minutes at room temperature. Slides were washed three times with PBST for 5 minutes each followed by incubation with Avidin-Biotin complex for 45 minutes at room temperature (VECTASTAIN UNIVERSAL Elite ABC Kit, PK-6200). Slides were rinsed two times with PBST for 5 minutes each, and then developed using 3,3’-diaminobenzidine (DAB) solution substrate-chromogen (Thermo Ultravision Plus, Thermo Scientific) and immediately immersed in distilled water to stop the reaction. Slides were counterstained with Hematoxylin QS (VECTOR) for 2 minutes and rinsed several times with distilled water. Finally, a coverslip was placed to overlay slides using SHUR/Mount (Electron Microscopy Services).

### Statistical analyses

One- and two-way ANOVA and Student’s t-tests were used for statistical analyses, as appropriate. Bonferroni post hoc was used, as appropriate. Significance was denoted as P < 0.05.

## Results

### LDH-A expression is inhibited using the Tet-Off system to express LDH-A shRNA

**Figure 2:**
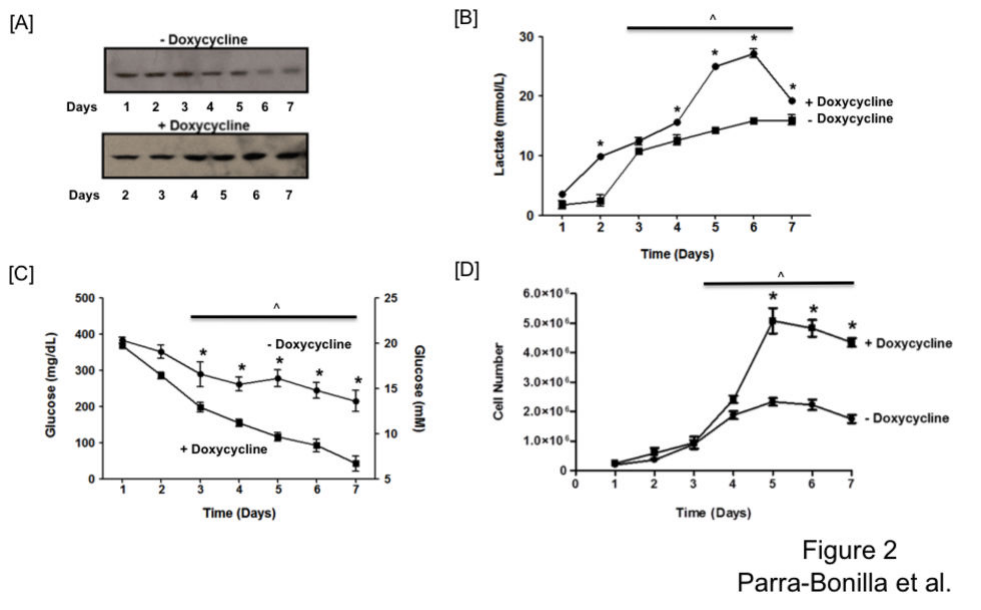
LDH-A is required for PMVECs to sustain aerobic glycolysis and rapid growth. Western blot analysis demonstrates that LDH-A protein decreases in the absence of doxycycline over a 7-day time course (A) resulting in decreased lactate production (B), glucose consumption (C) and PMVEC growth (D). Panel A shows a representative western blot taken from three different experiments performed in parallel with the growth curve. Two-way ANOVA was used to compare between groups, and Bonferroni post hoc test was performed as needed. Significant differences over time (^) and between doxycycline treatments (*) are shown (P < 0.05). Data represent averages + SEM from 3-5 experiments per group, each performed in triplicate.

We conditionally expressed LDH-A shRNA using a Tet-Off approach to confirm our previous results [13], that LDH-A is essential for PMVEC to sustain their glycolytic phenotype necessary for rapid proliferation, and to exclude an independent effect of doxycycline on cell growth. Tet-Off engineered cells were grown and expanded in the presence of doxycycline (2 µg/ml). Growth curves were performed with both induced (no doxycycline) and uninduced (doxycycline) cells. In induced cells, LDH-A levels progressively decreased throughout the growth curve. However, six days were required for LDH-A levels to dramatically decrease after doxycycline retrieval (Figure **2A** **, upper**). In contrast, in un-induced cells LDH-A levels remained relatively constant for the entire growth period (Figure **2A** **, bottom**). Lactate levels were decreased to half of that seen in uninduced cells (30 vs. 15 mM) (Figure **2B**), as was glucose consumption (Figure **2C**). Cell proliferation was inhibited in the induced cells (low LDH-A) (Figure **2D**), confirming our previous results using the Tet-On system [13].

**Figure 3:**
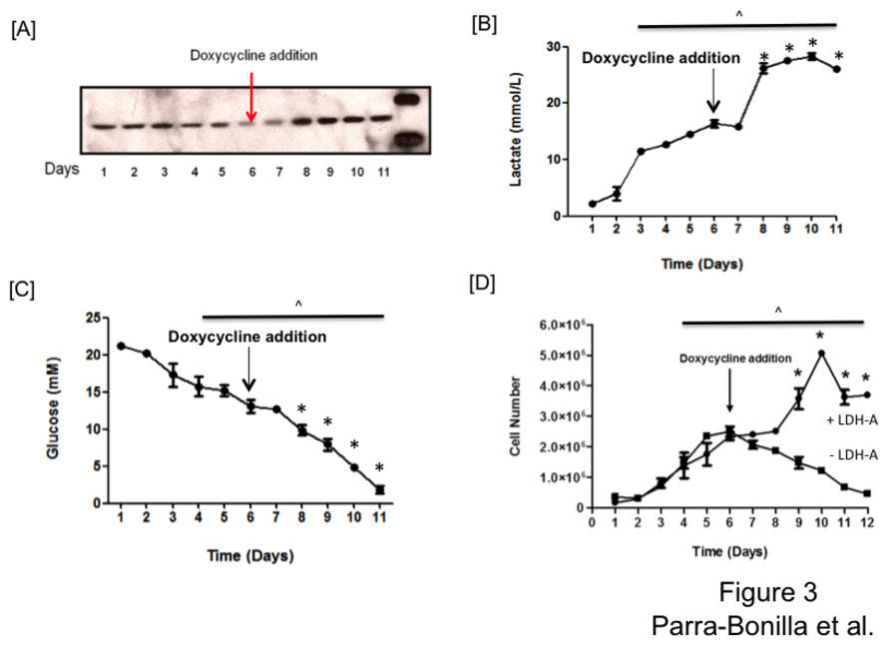
LDH-A rescue promotes PMVEC growth. Cells were grown in the absence of doxycycline for five days, and then on day six, doxycycline treatment was initiated. Doxycycline treatment rescued LDH-A protein (A), lactate production (B), glucose consumption (C), and PMVEC growth (D). One-way ANOVA was used to assess significance over the 12-day time course, two-way ANOVA was used to compare between groups, and Bonferroni post hoc test was performed as needed. Significant differences over time (^) and due to doxycycline treatment (*) are shown (P < 0.05). Data represent averages + SEM from 3-5 experiments per group, each performed in triplicate.

One advantage of the conditional expression system is that rescue experiments can be performed over time in the same cell population. Rescue experiments demonstrated that addition of doxycycline restored LDH-A expression (Figure **3A**), lactate production (Figure **3B**), glucose consumption (Figure **3C**) and PMVEC proliferation (Figure **3D**). These findings indicate that LDH-A expression is essential to the proliferative phenotype of PMVECs.

### LDH-A expression is necessary for network formation on Matrigel *in vitro*

**Figure 4:**
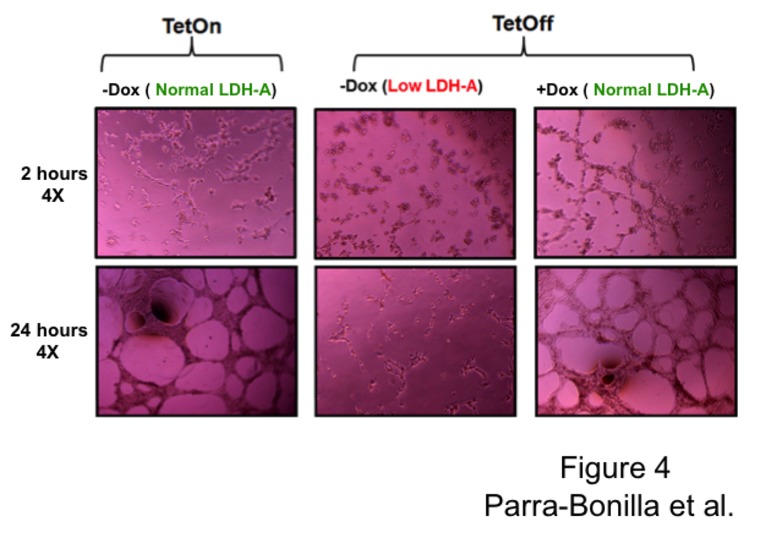
LDH-A is essential for network formation on Matrigel *in vitro*. 3x10^4^ cells from Tet-On and Tet-Off systems were seeded on Matrigel in 48 wells plate and kept at 37°C overnight. Cells expressing high LDH-A levels from both systems started to form networks as early as two hours after seeding, and complete networks were observed after 24 hours. Cells with low LDH-A levels failed to form networks.

To address the angiogenic potential of PMVECs *in vitro*, with normal and decreased LDH-A levels, a network formation assay was performed. Both the Tet-On and Tet-Off systems were tested. Tet-On cells grown in the absence of doxycycline and Tet-Off cells grown in the presence of doxycycline were considered to be controls, as both of these cell types express normal LDH-A levels. Tet-On cells grown in the presence of doxycycline and Tet-Off cells grown in the absence of doxycycline both express the LDH-A shRNA, and therefore possess low LDH-A levels. In control experiments, PMVECs grew from 1 x 10^5^ cells to 5 x 10^6^ cells over a seven-day time course, and networks started to form on Matrigel as early as 2 hours after cell seeding; 24 hours later networks were completely formed (Figure **4** **, LDH-A normal levels**). During the growth curve, PMVECs consumed glucose, generated lactate and decreased media pH (data not shown). In contrast, glucose consumption, lactate production and PMVEC growth were all attenuated following suppression of LDH-A expression in both systems. Most of the cells lacking LDH-A were not able to attach on Matrigel, and after 24 hours just irregular networks were formed (Figure **4** **, low LDH-A levels**). These results demonstrate that LDH-A is required for network formation on Matrigel *in vitro*, an approach that has been used as an indication of angiogenic potential.

### LDH-A expression is essential for neo-angiogenic capacity of PMVECs

The development of Tet-On and Tet-Off systems for the inducible expression of shRNA allows us to regulate LDH-A expression *in vivo* without the need to feed animals with doxycycline. We used Tet-On PMVECs to maintain normal LDH-A levels, and Tet-Off PMVECs to inhibit LDH-A expression; in this manner we were able to control LDH-A shRNA expression. In the first case, Tet-On PMVECs contain the shRNA, but the shRNA is not expressed if cells are not exposed to doxycycline (inducer). In contrast, Tet-Off PMVECs express the shRNA after doxycycline retrieval. We also tested a cell line engineered with the Tet-On system lacking LDH-A shRNA, and a second cell line engineered with the Tet-Off system lacking LDH-A shRNA. These two cell lines served as controls since they each express normal LDH-A levels. Wild type PMVECs were also used as controls. Cells were mixed with Matrigel, and subcutaneously injected into the abdomen of the rats.

**Figure 5:**
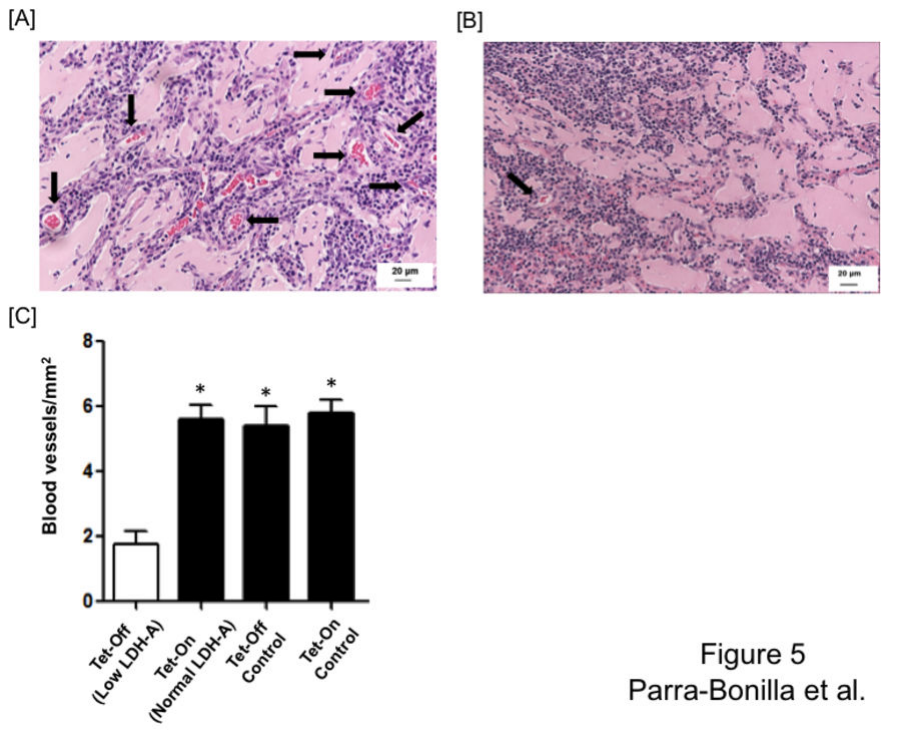
LDH-A expression promotes angiogenesis *in vivo*. Tet-On PMVECs expressing normal LDH-A levels or Tet-Off PMVECs expressing low LDH-A levels were mixed with Matrigel, and then injected in the abdomen of rats. Matrigel plugs were removed after 10 days and paraffin-embebeded slides were made and stained with hematoxylin and eosin for vessel counting. Representative micrographs show high density of vessel formation in Matrigel containing cells expressing normal LDH-A levels (A), and low density of vessel formation in Matrigel containing cells expressing low LDH-A levels (B). Arrows show blood vessels that formed within the Matrigel plug. (C) Vessel density assessment demonstrated that PMVECs expressing normal LDH-A levels (Tet-On, Tet-Off control and Tet-On control) form a significantly greater number of vessels than do PMVECs expressing low LDH-A levels (Tet-Off). One-way ANOVA and Bonferroni post hoc test was performed to assess significance of number of vessels formed by the different cell lines. *Significantly different p<0.05. Micrographs were taken at 20x. Arrows indicate vessels containing red blood cells.

After 10 days, we quantified the number of blood vessels in excised plugs that were formed by PMVECs expressing normal (Figure **5A**) and low LDH-A levels (Figure **5B**), as well as in control cells. Every lumen containing red blood cells was considered a vessel. We found that decreasing LDH-A expression in PMVECs decreased their angiogenic capacity by 3.2-fold when compared to PMVECs expressing normal LDH-A levels (Tet-On PMVECs) and control cell lines (Figure **5C**). The majority of vessels formed by PMVECs expressing normal LDH-A levels were capillary-like size, although bigger vessels were infrequently observed. We also noted some areas of hemorrhagic-like events in the plugs containing Tet-Off PMVECs (data not shown), which supports the idea of a failed attempt to generate a neo-angiogenic response in cells with low LDH-A protein levels.

**Figure 6:**
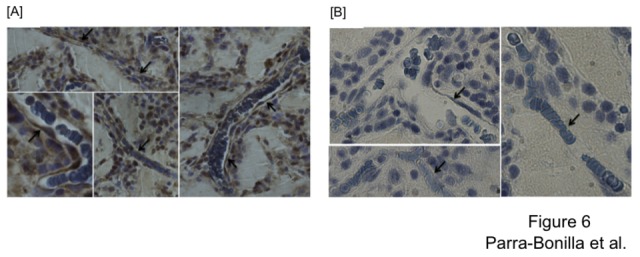
Cells injected in Matrigel are responsible for formation of new blood vessels. (A) Representative micrographs of GFP-positive endothelial cells. The presence of GFP was detected using anti-GFP antibody followed by biotinylated horse anti-rabbit secondary antibody, visualized using the DAB substrate system. Arrows highlight positive endothelial cell staining in intact vessels. (B) Micrographs depicting negative staining in rat sections using an isotype control antibody. Arrows indicate endothelial cells that do not display staining using the control antibody. All micrographs were taken using a Nikon 80i upright microscope fitted with a 60X objective.

Immunohistochemical analysis was performed using GFP antibody, which revealed that cells lining the formed vessels were indeed the injected PMVECs that contained the GFP sequence (Figure **6**). These results confirmed the *in vitro* observations shown in Figure **4**, where Tet-Off PMVECs were not able to form networks on Matrigel. Taken together, these *in vitro* and *in vivo* experiments indicate that LDH-A expression is essential to the neo-angiogenic capacity of PMVECs.

## Discussion

Proliferation is a fundamental property of living cells, although not all cells are similarly replication competent [15,16]. Endothelial cells isolated from the adult pulmonary circulation display variable growth potentials; PMVECs grow faster than do pulmonary artery endothelial cells [3,10,11,12]. Single cell cloning of these endothelial cell populations reveal progeny with high, intermediate and low proliferative potentials [10]. PMVEC populations can be comprised of 30-50% highly proliferative cells, whereas pulmonary artery endothelial cell populations are comprised of less than 10% of the highly proliferative cells. These findings suggest it is the replication competent cells that can be responsive to vascular injury in order to repopulate or repair the endothelium, and further, that the microcirculation is highly adapted to respond to injurious stimuli. Indeed, gene profiling of freshly isolated adult pulmonary capillary endothelium show expression of multiple genes characteristic of embryogenesis and vascular development [17]. Such cell growth may be necessary to generate new cells that line an existing blood vessel, may be necessary to increase the length of an existing vessel, or may be needed to generate *de novo* vessels within the postnatal circulation.

Angiogenesis and vasculogenesis are fundamental features of all endothelia. The ability to generate new blood vessels requires that existing endothelial cells proliferate as they build vascular tubes and establish connections with existing vessels conducting blood. Rapidly growing endothelial cells generate more blood vessels in angiogenesis assays. PMVECs are highly neo-angiogenic, generating 2-3 fold more blood vessels in a shorter time period when compared to pulmonary artery endothelial cells [10]. These findings suggest that the microcirculation is adapted to generate new blood vessels following vascular injury.

Rapid proliferation and neo-angiogenesis exert bioenergetic demands not typical for quiescent cells. Endothelial cells possess a relatively low number of mitochondria, and are not thought to possess high aerobic demands [3,18]. In response to growth-provoking stimuli, PMVECs utilize aerobic glycolysis to sustain the bioenergetic needs of rapid proliferation [13]. Aerobic glycolysis is defined by increased glycolytic flux with sufficient oxygen delivery to mitochondria, and is characterized by glucose consumption and production of lactate [19]. Glut-1 surface expression is necessary to transport glucose into the cell, hexokinase phosphorylates glucose in order to retain glucose in the cytoplasm, and LDH converts pyruvate into lactate regenerating NAD^+^ necessary to sustain glycolytic flux. Expression of each of these three enzymes is higher in PMVECs than in pulmonary artery endothelial cells, enabling PMVECs to generate 2-3 times higher ATP concentrations during growth [13]. LDH is a tetramer comprised of LDH-A and LDH-B proteins, which organize into one of five different combinations. The presence of LDH-A in the tetramer increases enzymatic activity, and is essential for PMVECs to display their rapid neo-angiogenic phenotype. Indeed, in the absence of LDH-A, PMVECs do not rapidly consume glucose, do not generate as much lactate, and do not rapidly proliferate relative to pulmonary artery endothelial cells.

In the present study, we sought to determine whether LDH-A was necessary for the neo-angiogenic capacity of PMVECs. To test this idea in both *in vitro* and *in vivo* angiogenesis assays, we developed a novel experimental system in which LDH-A could be conditionally silenced. PMVECs were generated for conditional expression of LDH-A shRNA using both Tet-On and Tet-Off approaches. Irrespective of whether Tet-On or Tet-Off approaches were used, expression of the LDH-A shRNA reduced LDH-A protein and decreased vascular tube formation *in vitro*. Using Tet-Off cell lines as a system in which the angiogenic potential of PMVECs lacking LDH-A could be tested in *in vivo* assays, we observed that LDH-A silencing similarly decreased blood vessel formation. We have interpretated these results to mean that aerobic glycolysis provided the ATP needed for neo-angiogenesis. However, we have not presently ruled out other possible contributing mechanisms. Lactate itself may be an important intracellular signal. LDH is directly involved in the redox status of the cell [20], and in gene regulation responsible for the metabolic switch that is necessary to accomplish demanding processes, such as neo-angiogenesis. LDH may form a protein complex consisting of the transcription factor oct-1 and its co-activator OCA-S, which is required for transcription of histone 2B (H2B). GAPDH and LDH are part of OCA-S, and even the enzymatic activity of LDH is necessary for the regulation of transcription of H2B [20]. Irrespective of the downstream mechanism, our present findings conclusively demonstrate the importance of LDH-A in establishing the bioenergetic demands for rapid endothelial cell neo-angiogenesis.

While our studies demonstrate that PMVECs display the intrinsic ability to rapidly generate new blood vessels, neo-angiogenesis of the pulmonary endothelium in the adult circulation *in vivo* has not been clearly demonstrated and remains controversial. This controversy may be partly due to the lung’s extensive vascular network, and the unique anatomy of the lung’s capillaries, which enable recruitment and distention and make it difficult to quantify neo-angiogenesis. These technical difficulties are compounded by the need to systematically separate angiogenesis of the lung’s systemic and pulmonary circulations. There is clear evidence that in airway inflammation [21,22,23] and pulmonary hypertension [24,25,26,27] the lung’s systemic blood supply undergoes a reversible angiogenic response. Further, following pulmonary artery ligation and lung ischemia the intercostal circulation invades the lung and forms an anastomosis with the pulmonary circulation [28]. In these instances, there is no evidence for angiogenesis of the pulmonary endothelium. However, in response to other, varied stimuli evidence has accumulated to suggest that pulmonary capillary endothelium undergoes angiogenesis, including: hepatopulmonary syndrome [26,29], post-transplant obliterative airway disease [30], pulmonary hypertension [31,32], *Pseudomonas aeruginosa* airway infection [33], pneumonectomy [34], cavopulmonary anastomosis [35], estrogen supplementation in ovarectomized rats [36], the early luteal phase of the menstrual cycle [36], and following L-arginine supplementation in pulmonary hypertension [37]. Provocatively, intra-tracheal delivery of hepatocyte growth factor [38,39], fibroblast growth factor-2 [40] and vascular endothelial cell growth factor [41] may induce angiogenesis and promote alveolar growth in lungs showing emphysematous changes. These reported angiogenic responses are beneficial to the organ, yet there are also examples of disordered angiogenesis in the setting of pulmonary hypertension, both in plexiform lesions of small precapillary vessels [42,43,44] and in pulmonary microvasculopathy of the capillaries [45]. The array of stimuli that appear to induce angiogenesis of pulmonary capillaries suggest this process is critically important in the adult circulation.

Angiogenesis is a fundamental property of endothelium, including in the lung, and especially lung microvascular endothelium. Expression of LDH-A is essential for sustaining aerobic glycolysis necessary to provide the bioenergetic demands of proliferation and angiogenesis. With increasing evidence that angiogenesis is an important biological phenomenon of the adult circulation, we must expand our understanding of the bioenergetic demands of angiogenesis in order to intercede in vascular injury and promote repair.
